# Relationship between Problematic Smartphone Use, Sleep Quality and Bedtime Procrastination: A Mediation Analysis

**DOI:** 10.3390/bs13100839

**Published:** 2023-10-13

**Authors:** Santiago Correa-Iriarte, Sergio Hidalgo-Fuentes, Manuel Martí-Vilar

**Affiliations:** 1 Departamento de Psicología Básica, Facultad de Psicología y Logopedia, Universitat de València, 46010 Valencia, Spain; coisan@alumni.uv.es (S.C.-I.); sergio.hidalgo@uv.es (S.H.-F.); 2 Departamento de Psicología y Salud, Facultad de Ciencias de la Salud y la Educación, Universidad a Distancia de Madrid (UDIMA), 28400 Madrid, Spain

**Keywords:** sleep quality, problematic smartphone use, bedtime procrastination, gender differences, age differences, mediation analysis

## Abstract

The purpose of this investigation was to study the relationship between sleep quality, problematic smartphone use (PSU) and bedtime procrastination, as well as to assess gender and age differences. A total of 313 participants, aged 18–60 (*M* = 30 ± 10.1; 53.2% males), completed an online survey between February and May 2023 in Spain. The Pittsburgh Sleep Quality Index, Smartphone Addiction Scale-Short Version and Bedtime Procrastination Scale were used to measure sleep quality, PSU and bedtime procrastination, respectively. Additionally, smartphone use habits were evaluated through self-report questions. Pearson correlations, independent samples *t*-tests, one-way ANOVA, Bonferroni’s post hoc tests and mediation analysis were conducted. Correlation analysis showed positive associations between the three main variables. Independent sample t-tests indicated females were more prone to PSU along with higher overall smartphone use. Post hoc analysis of one-way ANOVA exposed age differences between young adults (18–25 years old), adults (26–44 years old) and middle-aged adults (45–60 years old) in PSU and bedtime procrastination. Finally, mediation analysis revealed that PSU had indirect effects on sleep quality through bedtime procrastination, but no direct effects on sleep quality. Therefore, PSU, and especially bedtime procrastination, should be considered as targets in future campaigns or intervention programs to improve sleep quality among the young Spanish population.

## 1. Introduction

Sleep quality is a person’s subjective assessment of how well they feel they have slept [1]. In recent years, there has been growing concern about sleep-related problems (e.g., sleep efficiency, sleep latency or sleep quality) due to the importance of sleep in the overall health of the population, especially in mental health [2]. According to the Spanish Society of Neurology [3], more than 10% of the Spanish population will suffer from a severe chronic sleep disorder. In addition, 20–48% of Spanish adults and 20–25% of children have some difficulty initiating or maintaining sleep. There is no consensus on a gender difference in sleep quality, with some studies claiming that women have poorer sleep quality [4,5,6], while others do not find this relationship [7,8].

According to Stahl [9], disturbances in sleep–wake cycles are associated with an increase in mental disorders (e.g., major depression or anxiety disorders); immune system, cardiovascular and metabolic disorders (e.g., diabetes or stroke); neurological disorders (e.g., Alzheimer’s dementia or chronic pain); endocrine dysfunction (e.g., in the hypothalamic–pituitary–adrenal axis); cancer; and other derived economic costs (e.g., loss of productivity or cost of accident repair).

Considering the health risks associated with sleep disturbances, data on the increase in sleep problems since the global COVID-19 pandemic may be cause for concern. A study of 19,267 adults in 13 Asian, American and European countries [10] found a significant increase in sleep and mental health problems since the COVID-19 pandemic. Similar results have been found in studies conducted in China [11], where the prevalence of clinical insomnia has increased by 37% since the COVID-19 pandemic, or in Spain, where 23.9% of a sample of 15,070 people reported having problems initiating or maintaining sleep [12]. Considering that during the confinements the amount of time spent in front of screens increased for people of all age ranges [13], many health professionals have been interested in whether there is a link between increased screen use and sleep problems.

This becomes even more relevant when considering the widespread use of smartphones in the population and their suitability for use in bed [14]. In the case of Spain, and according to data from the National Institute of Statistics (INE) [15], 99.2% of people aged 16–74 used smartphones in the three months prior to the survey (conducted in November 2022). Smartme Analytics [16] reflects in its latest report that Spanish adults use, on average, a smartphone for 3 h and 40 min, a figure that increases to 4 h and 15 min a day in the case of young people between 18 and 24 years old.

Problematic smartphone use (PSU) is defined as the use of a smartphone associated with at least some element of dysfunctional use, such as anxiety when the smartphone is not available or neglect of other activities due to smartphone use [17]. These negative or dysfunctional effects can range from withdrawal to loss of control over phone use, decreased productivity, impaired daily functioning, detriment to social relationships or damage to physical health [18,19,20,21]. PSU is closely related, or even overlapping, with other phenomena such as problematic use of social networks, messaging apps or the internet [22,23,24]. However, there are some differences in terms of risk factors, for example, men tend to develop more problematic internet use, while women are more at risk of manifesting PSU [25]. The terminology related to behavioural addictions when researching smartphones is controversial, as some authors think that this may stigmatise smartphone users [21]. Moreover, PSU is not listed as an addiction in any of the main diagnostic manuals, neither the DSM-5 [26] nor the more recent ICD-11 [27]. Thus, in addition to “problematic smartphone use” and “smartphone addiction” other terms have been used to describe this type of relationship with smartphones: “excessive use”, “compulsive use” and “compensatory use” [28,29,30].

The prevalence of PSU in adults varies in different countries, for example, in Arabia it is 66.9% [31], in Bangladesh 61.4% [32] and in China estimates range from 65.8 to 52.8% [33]. Likewise, in Spain, several studies have placed the prevalence of PSU between 20.5 and 23.75% [34,35]. It is worth noting the difficulty in assessing and comparing the prevalence of PSU due to the inconsistency of diagnostic criteria and assessment methods [36]. Additionally, although some studies have found no sex differences [37], there is some consensus in the scientific evidence that women are at higher risk of developing PSU [38,39,40,41,42,43,44,45]. These differences could be caused by a different pattern of smartphone use, with women using smartphones for social purposes (i.e., social networking or instant messaging) and men for more varied purposes, such as video games, calls, and multimedia content [46,47]. In addition, women (especially younger women) may have a higher prevalence of PSU due to having more malleable and influenceable self-control in social situations than men [48,49].

Several negative consequences of PSU have been found, such as low productivity [50], poor academic performance [51,52], general procrastination [53], academic procrastination [54], low self-esteem [55], increased alcohol consumption [52], anxiety and depression [56,57,58], executive function deficiencies [59,60] and sleep problems [61,62,63,64,65].

### 1.1. Mechanisms by Which Smartphone Use Affects Sleep

#### 1.1.1. Disruption of Circadian Rhythms

First, it has been proposed that smartphone use close to sleep time may alter the production of melatonin and/or cortisol, both of which are important hormones in the regulation of circadian rhythms.

Melatonin is a hormone secreted by the pineal gland and regulated by sleep-inducing light/dark cycles. The pineal gland is a neural structure related to the visual system and has retinohypothalamic connections with the suprachiasmatic nuclei that house the internal biological clock and play a crucial role in generating circadian rhythms. It has been shown that light exposure in the evening can delay the phase of the internal clock, resulting in sleep problems, while light exposure in the morning advances melatonin secretion [66].

Cortisol, on the other hand, is a steroid hormone produced in the adrenal cortex, related to sleep arousal and wakefulness [67]. Cortisol shows a circadian rhythm, with a peak at the transition between sleep and wakefulness. After awakening, secretion of the hormone decreases throughout the day, reaching a minimum around midnight, before gradually increasing again to reach a new peak the following morning. Thus, cortisol, like melatonin, serves as a marker of an organism’s circadian temporal structure, regulating the sleep/wake cycle.

LED-backlit displays (such as smartphones) emit 3.3 times more light in the blue range (440–470 nm) than non-LED-backlit displays (e.g., some eReaders or displays using cathode ray tubes), as reported by Cajochen et al. [68] This difference is relevant as studies indicate that human circadian physiology and alertness levels are particularly sensitive to short-wavelength light [69,70,71,72].

Night-time exposure to an LED-backlit computer screen has been shown to cause a decrease in salivary melatonin levels [69] and an increase in waking time, along with improved cognitive performance, sustained attention, and working and declarative memory [68,73]. However, any delay in the circadian release of melatonin has negative consequences for sleep induction [74]. Schmid et al. [75] compared the effect on melatonin, cortisol and sleep levels of reading before sleep on a smartphone versus reading a printed book. They found that melatonin and cortisol levels were found to be altered in the smartphone use condition, in addition to a reduction in slow wave sleep. According to a study by Wallenius et al. [76], school children who used digital media for three hours a day showed a decrease in the cortisol increase one hour after waking up, showing an alteration in the circadian rhythm that this hormone follows. In contrast, children who used digital media for less than three hours or not at all showed a typical increase in cortisol in the morning. Another study with children showed a greater increase in cortisol just after using DVD screens versus playing with wooden blocks [77].

It is worth noting that the closer-to-face use of smartphones compared to other traditional media such as television [78], which is usually placed at a greater distance from the face, can lead to greater exposure to shortwave light [79] and thus cause greater sleep disturbance compared to other types of screens. In fact, a study by Figueiro et al. [80] found that the light emitted by 70-inch LED-backlit LCD televisions located 1.8 to 2.7 metres from the subjects did not alter melatonin production in adults.

Regarding radiofrequency electromagnetic fields (RF-EMFs) emitted by smartphones, a review of the literature by Selmaoui and Touitou [81] states that no conclusive evidence has been found that this type of radiation alters melatonin or cortisol secretion. Instead, according to these authors, there are indications that melatonin may be a protective agent against the negative effects of RF-EMFs, such as oxidative stress and DNA damage, as well as having neuroprotective properties.

#### 1.1.2. Increased Arousal

According to another line of research, increased arousal (especially cognitive and somatic arousal) before sleep may negatively influence sleep quality.

A study by Kheirinejad et al. [82] used the OURA wearable [83] to measure different components of sleep and the AWARE instrument [84] to assess smartphone usage by automatically collecting usage data. They concluded that the cognitive activation required during bedtime to perform different smartphone uses, such as conversing with other people or consuming images, text, video and audio, have a negative impact on sleep quality, but without a significant difference between them. On the other hand, Ong et al. [85] proposed a model in which two types of cognitive arousal, primary and secondary, contribute to the maintenance of insomnia. Secondary (metacognitive) cognitive arousal would encompass biases towards sleep-related thoughts and behaviours, rigidity in behavioural or sleep-related beliefs, and absorption in sleep problem solving. Primary cognitive arousal would consist of expectations about sleep, beliefs about the daytime consequences of sleep deprivation and, what concerns us in this section, increased mental activity at bedtime.

One of the main uses of smartphones is social media and communication applications, which sometimes require high cognitive functioning, which is not suitable for sleep induction or good sleep quality [86]. Specifically, of the 3 h and 40 min of smartphone time per day of Spanish adults, 1 h and 20 min are spent on social networks and another 40 min on instant messaging applications [16], which combined account for more than 50% of the total time spent on smartphones. It has been shown how increased arousal before sleep is a mediating variable between the negative effect of binge-watching TV series via smartphones [87] or the use of social networks [88] on sleep quality.

However, although correlational studies point to increased arousal as a possible cause of reduced sleep quality [89,90,91], an experimental study blocking the effect of blue light from smartphones conducted by Combertaldi et al. [92] found no such relationship. They did not observe empirical evidence of increased arousal from the use of social networks such as WhatsApp or Snapchat, nor a reduction in sleep quality. The authors hypothesise that the negative effect of smartphone use on sleep quality is due to the use of smartphones at bedtime (which usually exceeds 30 min) and its corresponding displacement of sleep time.

#### 1.1.3. Bedtime Procrastination and Sleep Displacement

Self-regulation, according to Gillebaart [93], can be defined as the cognitive ability to monitor, plan and guide a person’s behaviour to facilitate goal attainment and inhibit disruptive emotions and behaviour. Proper self-regulation requires adequate functioning in the brain’s reward system and top-down control of the prefrontal cortex [94]. As Zhang and Wu [95] point out, it has been shown that addictive behaviours can alter brain circuits related to self-regulation such as prefrontal cortex functioning and top-down control [96,97,98,99,100]. A deficit in inhibitory control, a feature closely related to self-regulation, is present in individuals with a PSU [101]. Additionally, Rebetez et al. [102] suggested the depletion of self-regulatory resources and the failure of self-regulation as a source of procrastination. Thus, when talking about self-regulation and sleep, we must talk about bedtime procrastination, a relatively recent concept [103] that is defined as the action by which people deliberately delay going to bed without external interference, even though negative outcomes are anticipated.

A qualitative study by Nauts et al. [104] found three reasons why people procrastinate sleep: deliberate bedtime procrastination, unconscious bedtime procrastination and planned delay. The first refers to consciously delaying sleep time to perform tasks that could be done at another time or to have a moment to oneself after a long day of work. Unconscious bedtime procrastination occurs when, for example, people lose perception of time while absorbed in a task. Finally, strategic procrastination is when people make a conscious decision to delay sleep in order to avoid negative emotions related to rumination, long sleep latency or as a remedy for insomnia (accumulating “sleep pressure”). However, some authors argue that the belief held by these subjects that sleep delay benefits them (even if it does not) means that strategic delay is not considered bedtime procrastination per se [104,105,106].

Kroese and De Ridder [106] indicate how people with low self-regulation show higher bedtime procrastination as well as insufficient sleep. Another study by Ma et al. [107] involving 1550 university students found that bedtime procrastination is a strong predictor of prevalence and severity of poor sleep quality. Authors have proposed that smartphone use may be one of the causes of the so-called displacement theory. This theory is based on the idea that unstructured leisure use of electronic devices, such as smartphones, can displace other activities, such as sleep. Thus, smartphone use may delay sleep time (causing bedtime procrastination) and possibly reduce the amount of sleep, or even create an association between being in bed and being active [108,109,110]. In this regard, a study conducted in China involving 2741 university students found that a total of 57.5% of their sample used a smartphone in bed [111]. At the same time, a Danish study [112] found that 12% of participants used their smartphone for 3–5 h late at night. There is no consensus regarding gender differences in bedtime procrastination, as some studies find significant differences [113], while others do not [114].

In addition, procrastination using the smartphone can induce negative self-evaluations such as self-defeating thoughts [115]. Consequently, these self-evaluations may in turn cause stress [116], guilt [117] or feelings of self-condemnation [118], which manifest as sleep problems [115]. The same can be inferred to be true for bedtime procrastination, as it is a form of procrastination and has been directly linked to depression [119]. The opposite relationship has also been observed, where rumination and other forms of negative affect may increase bedtime procrastination and thus affect sleep quality [120]. Moreover, some studies suggest that smartphone use may be a form of experiential avoidance of negative emotions [121]. This could be explained by the bidirectional relationship between PSU, increased depressive or anxious symptoms, and vice versa [122,123]. In addition, it has also been shown that the ability of smartphones to impair sleep quality and sleep drift can increase symptoms of depression and stress [124]. Simultaneously, PSU itself may increase depression and anxiety, and thus impair sleep [61]. This impact could form a vicious cycle in which negative emotions (including depressive and anxious symptoms towards sleep quality or bedtime procrastination) lead to maintaining or increasing their smartphone use and fuel negative affectivity.

Recently, different authors have conducted different studies based on mediational analyses observing how the impact of smartphone use (problematic or not) in sleep quality is mediated by bedtime procrastination, in addition to other variables such as self-regulation [95], psychological detachment [125] or fear of missing out (or FoMO) [126]. A similar phenomenon has been observed in the impact of problematic internet use and poorer sleep quality, mediated by bedtime procrastination [127].

**Figure 1:**
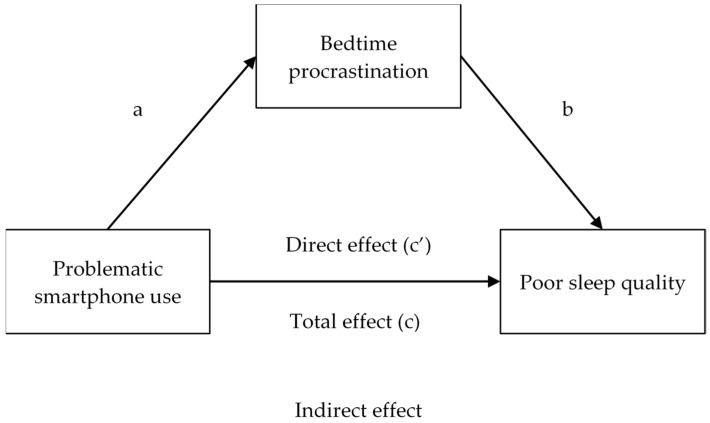
Hypothetical mediation model of bedtime procrastination between PSU and poor sleep quality.

Given this information, we propose the following research questions:

RQ1: To what extent do Spanish adults have problems with sleep quality and bedtime procrastination, and make intensive use of smartphones (throughout the day and before going to sleep)?; RQ2: Are there gender differences in the above variables?; RQ3: Are there age differences in PSU, bedtime procrastination and sleep quality?; RQ4: Is it possible that bedtime procrastination has a mediational effect between PSU and sleep quality (as seen in Figure 1)?

## 2. Materials and Methods

### 2.1. Participants and Procedure

A total of 313 people participated in the study, between February and May 2023, using non-probability convenience sampling. Three participants with outliers, due to input errors (e.g., 26 h of sleep or 288 years using smartphones), were excluded, giving a final total of 310 subjects. All participants, who ranged in age from 18 to 60 years (*M* = 30; *SD* = 10.1; 46.8% female and 53.2% male), completed a questionnaire anonymously and without compensation via the online questionnaire platform Google Forms. After explaining the aim and scope of the study, the questionnaire included a specific item asking for the consent of the participants, informing them that their answers would be anonymous and only accessible by the researchers, and that the results would be displayed in an aggregated and anonymous form. The questionnaire was disseminated via the social networks WhatsApp and Instagram.

### 2.2. Instruments

#### 2.2.1. Sleep Quality

The Pittsburgh Sleep Quality Index (PSQI) [128] includes 19 individual items about the subject’s sleep in the last month, divided into 7 components: subjective sleep quality (1 item), sleep latency (2 items), sleep duration (1 item), habitual sleep efficiency (3 items), sleep disturbances (9 items), use of sleep medication (1 item) and dysfunction during wakefulness (2 items). Each item is rated on a scale of 0–3, 0 being no difficulty and 3 being severe difficulty. Thus, the total score of the scale results from all components ranges from 0 to 21, where a higher score means worse sleep quality. In the original version, it is described that scores below 5 would be translated as “no sleep problems”, from 5 to 7 “deserves medical attention”, from 8 to 14 “deserves medical attention and treatment” and scores above 15 “severe sleep problems”. However, the Spanish validation used in the present study by Macías-Fernández and Royuela-Rico [129] only distinguishes between scores equal to or lower than 5 as “good sleep quality” and higher than 5 as “poor sleep quality”. In this study, the internal consistency of the PSQI was α = 0.69.

#### 2.2.2. Bedtime Procrastination

To assess bedtime procrastination, the Bedtime Procrastination Scale (BPS), originally created by Kroese et al. [103], was used. It is especially used in research on sleep deprivation and health problems, as indicated by the authors of the translation and validation of the scale in the Spanish population [130]. It contains a total of 9 items. The items are scored on a Likert scale from 1 (almost never) to 5 (almost always). The scale ranges from 9 to 45. A higher score is a sign of greater bedtime procrastination. In this study, the reliability achieved by the BPS was α = 0.84.

#### 2.2.3. Problematic Smartphone Use

PSU was measured with the Smartphone Addiction Scale-Short Version (SAS-SV) [17], a shortened version of the Smartphone Addiction Scale (SAS), developed by Kwon [131]. The SAS-SV consists of 10 items. These items are scored on a Likert scale from 1 (strongly disagree) to 6 (strongly agree), giving a total range of 10 to 60 points, with a higher score interpreted as a higher PSU. The original version placed the cut-off point for the PSU at 31 for women and 33 for men. In the Spanish validation of the SAS-SV questionnaire, conducted by Lopez-Feranandez [132], no gender differences were found, so 32 was chosen as the cut-off point for both genders. The reliability of the SAS-SV for this study was α = 0.85.

#### 2.2.4. Smartphone Usage Pattern

In addition to the standardised tests, information was also collected on estimated daily smartphone use, smartphone use in bed and how long they had owned a smartphone, by means of an ad hoc questionnaire. To assess smartphone use in bed, 4 questions were asked: whether they used their smartphone in bed (yes/no), how many days a week they used their smartphone in bed, for how long (less than 15 min, 16–30 min, 31–60 min or more than 60 min) and, finally, what activity they did with their smartphone in bed (calling, texting, surfing the internet, checking social networks, watching multimedia content, playing video games or other).

### 2.3. Statistical Analysis

The sample size was calculated with the G*Power tool of Faul et al. [133] for a mean effect size, an alpha of 0.05 and a statistical power of 0.99 with two predictors, obtaining a minimum of 125 participants. For the rest of the analyses, the statistical package IBM SPSS for MacOS (Version 25, Chicago, IL, USA) was used. The internal consistency of the tests used was analysed using Cronbach’s alpha and descriptive statistics were calculated for the sociodemographic variables and the study variables. Normal distribution of the sample was analysed using skewness and kurtosis statistics for the different variables, which were found to be below ±1, indicating relatively normal distributions according to the criterion proposed by Bulmer [134]. Pearson’s correlation was used to examine the association between the variables under study.

In addition, independent sample t-tests were performed to compare means and analyse differences between genders. Effect size was calculated following Cohen’s *d* statistic, which was interpreted as follows: 0.2, small; 0.5, medium; and 0.8, large. One-way ANOVA was performed to compare the scores of the sleep quality, PSU and bedtime procrastination variables according to different age groups: young adults (aged 18–25 years), adults (aged 26–44 years) and middle-aged adults (aged 46–60 years). Additionally, the effect size of the one-way ANOVA was estimated using the η2 statistic, with a value of 0.01, 0.06 and 0.14 being a small, medium and large effect, respectively. To check which groups were different, post hoc comparisons were performed using the Bonferroni test.

Moreover, we employed the 5000-sample bootstrapping method of Hayes [135] using the PROCESS macro (version 4.2 for SPSS) to perform a simple mediation analysis and test the possible mediational effect of bedtime procrastination on the effect of PSU on sleep quality. We calculated the effects of PSU on bedtime procrastination (path a), of bedtime procrastination on sleep quality (path b) and direct effect of PSU on sleep quality (path c’). In addition, we analysed the indirect effect (path a*b) of the PSU on sleep quality through bedtime procrastination, and finally the total effect of the model (path c), the sum of paths c’ and a*b. The coefficients of the direct and indirect effects were calculated with a 95% confidence interval (CI).

## 3. Results

### 3.1. Descriptive Statistics

Socio-demographic characteristics of the participants can be found in Table 1. It is worth noting that 79% of the sample used the smartphone in bed and, of these, 16% used it for less than 15 min, 34.3% for 16–30 min, 34% for 31–60 min and 15.6% for more than 60 min.

Table 2 shows the descriptive statistics of the participants for the study variables. Regarding sleep quality, the overall mean of the participants was within the “requires medical attention” range proposed by the original PSQI, and in the “poor sleepers” category of the Spanish validation (*M* = 7.5, *SD* = 3.6). Mean PSU and bedtime procrastination were *M* = 28.9, *SD* = 10.0 and *M* = 25.2, *SD* = 3.7, respectively. Participants owned a smartphone for 11.9 years on average (*SD* = 4.3) and used it 5 h per day (*SD* = 2.8). In addition, they used the smartphone on an average of 5.9 days per week (*SD* = 1.9). The variable of time spent using the smartphone in bed is scored using a Likert-type scale (*M* = 2.5, *SD* = 0.9) and has a median of 3 (2–3).

Overall, 31.6% of the sample had good sleep quality compared to 68.4% who had poor sleep quality. With regard to the PSU, 37.2% of the participants showed signs of a PSU as measured by the SAS-SV scale, while 59.7% did not show these characteristics. A total of 69.7% of the women in the sample had a PSU, compared to 67.3% of the men.

### 3.2. Correlations

As shown in Table 3, statistically significant correlations between the three main study variables were found. The strongest correlation between these three is between PSU and bedtime procrastination. Moreover, poor sleep quality correlates positively with higher PSU and higher bedtime procrastination. In addition, spending more hours on the smartphone is also positively correlated with poorer sleep quality, PSU and bedtime procrastination. We found a positive correlation between a greater number of days using a smartphone in bed and a higher PSU, higher bedtime procrastination and higher smartphone overall use. Regarding the time of smartphone use during bedtime, this correlates positively with poorer sleep quality, higher PSU, higher bedtime procrastination, more daily smartphone hours and more days per week using smartphones in bed. We observed a negative correlation between more years of smartphone use and more time using smartphones during bedtime. Older age is negatively correlated with PSU, bedtime procrastination, daily hours of smartphone use and smartphone use in bed. However, no significant correlation was found between age and sleep quality.

### 3.3. Mean Comparison between Genders

As can be seen in Table 4, differences were found between men and women in PSU (*t*_(308)_ = 3.248, *p* = 0.001). However, no differences were found in sleep quality (*t*_(308)_ = 0.210, *p* = 0.834) and bedtime procrastination (*t*_(308)_ = 1.031, *p* = 0.303). Finally, we observed that women used a smartphone for 1.1 h more on average per day than men (*t*_(306)_ = 3.115, *p* = 0.002), while men owned smartphones for 1.4 years more than women (*t*_(306)_ = −2.311, *p* = 0.021). For all statistically significant differences, the effect size is small.

### 3.4. Comparison between Age Groups

Results of one-way ANOVA are shown in Table 5. The ANOVA for sleep quality showed that there were no significant differences between any of the age groups (*F*_(2, 306)_ = 0.60, *p* = 0.548). However, statistically significant differences were found for PSU (*F*_(2, 306)_ = 8.73, *p* < 0.001) and bedtime procrastination (*F*_(2, 306)_ = 7.15, *p* = 0.001), with a small, close to medium effect size (η2 = 0.05).

Bonferroni post hoc contrasts (Table 6) show that mean problematic smartphone use is significantly different (*p* < 0.05) between young adults (*M* = 31.6, *SD* = 9.5) and adults (*M* = 27.6, *SD* = 10.3), and between young adults and middle-aged adults (*M* = 25.4, *SD* = 9.3). There were no statistically significant differences between adults and middle-aged adults (*p* > 0.05). Similarly, we found statistically significant differences (*p* < 0.05) in mean bedtime procrastination between young adults (*M* = 30.7, *SD* = 7.6) and adults (*M* = 28.3, *SD* = 7.0), and between young adults and middle-aged adults (*M* = 26.2, *SD* = 6.3). We found no significant differences between adults and middle-aged adults (*p* > 0.05).

### 3.5. Mediation Analysis

**Figure 2:**
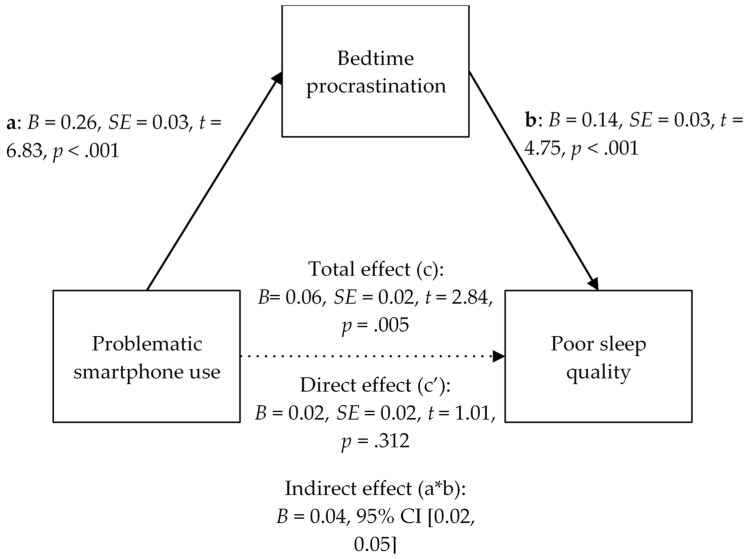
Mediation model of bedtime procrastination between PSU and poor sleep quality. Note. The dashed line indicates a non-significant direct effect (*p* > 0.05).

As for the results of the mediation analysis, as can be seen in Table 7 and Figure 2, there is a statistically significant effect between PSU and bedtime procrastination (path a; *B* = 0.26, *SE* = 0.03, *t* = 6.83, *p* < 0.001), and between bedtime procrastination and worse sleep quality (path b; *B* = 0.14, *SE* = 0.03, *t* = 4.75, *p* < 0.001). Likewise, an indirect effect (i.e., the effect of PSU on sleep quality through bedtime procrastination) was found to be significant (*B* = 0.04, 95% CI [0.02, 0.05]). However, the direct effect of PSU on sleep quality is not significant (*B* = 0.02, *SE* = 0.02, *t* = 1.01, *p* = 0.312). Finally, we found a significant overall effect between PSU and sleep quality (*B* = 0.06, *SE* = 0.02, *t* = 2.84, *p* = 0.005), taking into account any indirect effect through bedtime procrastination. Therefore, we can conclude that bedtime procrastination has a full mediation effect (the direct effect c’ is not significant) on the impact of PSU on poorer sleep quality.

## 4. Discussion

Our study aimed to describe data on sleep quality problems, smartphone use (in general and during bedtime), PSU and bedtime procrastination in Spanish adults (RQ1); analyse possible gender differences in the variables mentioned above (RQ2); study disparities between age groups in terms of PSU, bedtime procrastination and sleep quality (RQ3); and, finally, analyse a possible mediational effect of bedtime procrastination on the relationship between PSU and sleep quality.

Thus, in terms of RQ1, we found that the majority (68.4%) of the sample had poor sleep quality, and that more than a third (37.2%) showed signs of presenting a PSU; although these are higher numbers, they are not very far from other studies involving Spanish adults [34,35]. Additionally, participants reported a daily smartphone usage of 5 h, which is 1 h and 20 min higher than the data from Smartme Analytics [16] in Spain. Furthermore, we observed that more Spanish adults use their smartphone in bed (78.9%) than in other countries such as China, where 57.5% use it [111]. Furthermore, 15.6% of the sample displaced their sleep time by more than one hour by using their smartphone in bed.

On the other hand, and responding to RQ2, we can see that women tend to use their smartphones for longer periods of time, which is one of the risk factors for PSU. Moreover, they show a higher PSU, which is consistent with numerous investigations [25,38,39,40,41,43,44,45]. As indicated by different authors [46,47], women’s smartphone use is predominantly social (e.g., social networking or instant messaging), compared to a more diverse pattern for men. Greater use of social networks has been found to be associated with a detriment in the perception of real social support [136]. Thus, it is possible that, by making their smartphone use pattern more social, women may feel less real social support and try to mitigate this by increasing their use of social networks, resulting in higher PSU. Additionally, we found no gender differences in bedtime procrastination, similar to previous research [113], but contradicting the direct effect of PSU on bedtime procrastination proposed by this research. It is possible that another unstudied variable is involved in this relationship, for example, greater responsibility in women [137]. As there are no differences in bedtime procrastination, according to the mediational model of this study that we will detail later, the finding of no differences in sleep quality is consistent with other research [7,8].

In relation to RQ3, we found differences in the PSU and bedtime procrastination variables in young adults (18–25 years) versus adults (24–44) and middle-aged adults (45–60), but no differences in sleep quality. The differences in PSU are consistent with the literature [138], and are possibly due to the fact that younger people spend more hours on their smartphone (both variables correlate significantly, as can be seen in Table 3) and that younger generations have grown up with this technology [139]. Regarding bedtime procrastination, these results are also consistent with previous research [140]. It is likely that young adults, having a higher PSU, delay tasks they have to perform during the day, and thus procrastinate at their sleep time as well. However, young adults, who show higher PSU and bedtime procrastination, do not show worse sleep quality. This result seems to be inconsistent with the indirect effect of PSU on sleep quality, mediated by bedtime procrastination, as proposed by the model of this study and discussed in RQ4. This may be due to the fact that a large proportion of young adults are still studying (85.1% of the sample), so they have greater flexibility in their schedules to compensate for a shift in sleep time caused by the PSU, for example, by not attending classes the morning after having procrastinated sleep or by sleeping more on days off (sleep duration is one of the components of the PSQI scale). Adults, being mostly workers (79.4% of the sample), generally have less flexible schedules and are therefore less able to compensate for sleep displacement. In fact, a study comparing sleep parameters of non-working versus working high school students found that the latter were sleepier during the day [141]. In addition, adults and middle-aged adults may generally have increased stress or health problems (e.g., pain or respiratory diseases) that affect sleep, apart from PSU and bedtime procrastination.

As for our RQ4, the mediation analysis shows that PSU is not directly related to poorer sleep quality, but affects sleep quality through the indirect effect of bedtime procrastination (i.e., the mediation of bedtime procrastination is total). These results are in agreement with previous research [125], and especially with Zhang and Wu [95], as they also found no direct relationship between PSU and sleep quality, unlike Huang et al. [126]. Considering the reasons for bedtime procrastination described by Nauts et al. [104], two possible causes of bedtime procrastination related to higher PSU may be receiving the gratification of taking time for oneself using the smartphone, or the loss of a sense of time when consuming content on smartphones. The latter possibly is supported by the nature of many smartphone apps, which are designed to retain the viewer as long as possible and form consumption habits [142]. In addition, as we have discussed above, higher PSU may cause the delay of completing activities during the day, as it has been related to general [58] and academic [54] procrastination, which would cause people to perform postponed tasks throughout the day and during the night, increasing bedtime procrastination, shifting sleep time and thus affecting sleep quality. Furthermore, as described above, PSU can elicit negative affectivity [115,116,117,118] that feeds back on itself by negative feelings due to bedtime procrastination and poor sleep quality [122,123], thus sustaining these phenomena.

## 5. Conclusions

Although gender and age differences in PSU had small effect sizes, these results may help to identify the population profiles most vulnerable to PSU and its negative effects, especially considering the novelty of the topic and the lack of a total consensus regarding gender differences in the literature. The practical utility of detecting gender differences in PSU is observed in publications by other authors, which suggest the need to develop gender-specific interventions. More specifically, training in self-awareness and self-control in adolescent females has been proposed [143]. On the other hand, results of age differences not only let us know that younger adults have a higher risk of PSU, but also suggest the existence of possible distinct PSU effects in different age groups [144]. Younger adults with PSU tend to show greater interpersonal and intrapersonal conflicts than older adults. The latter also show fewer physical and psychological withdrawal symptoms, but these are of greater weight. Taking into account these age and gender differences may be helpful in designing more tailored interventions to each population profile.

From this study we conclude that the smartphone is a source of problematic use (especially for young people and women), which can displace users’ sleep through bedtime procrastination (consciously or not) and thus negatively impact sleep quality. To our knowledge, no study has been carried out involving the Spanish population exploring gender and age differences in bedtime procrastination. Nor are we aware of any research carried out involving Spanish adults that relates PSU, sleep quality and bedtime procrastination (in addition to its mediational effect on the relationship between the first two).

Finally, we must highlight the limitations of the present study. Firstly, the cross-sectional design and the accidental non-probabilistic sampling limit the generalisability of the results. On the other hand, the measurement of the variables, being based on self-report tests, may not fully conform to reality (e.g., due to social desirability effects or difficulties in understanding the questions), although the anonymous and voluntary nature of the study minimises this risk. Furthermore, the effect of other latent variables (such as self-regulation or rumination) or reciprocal relationships between the study variables have not been explored. Thus, future studies could consider a more complex analysis (e.g., based on structural equation modelling), controlling for possible confounding factors by including demographic variables such as socioeconomic status in the analysis, and a longitudinal design and random sampling, so that causal relationships between these variables could be established in the Spanish population, something that has not been done to date.

The above results and conclusions point to the PSU, and especially to bedtime procrastination, as potential treatment targets to improve sleep quality. Thus, efforts could be made to raise awareness among the Spanish population (especially women and young adults) and to try to implement interventions, such as smartphone apps, that prevent the negative effects of PSU, bedtime procrastination and poor sleep quality.

## Figures and Tables

**Table 1:** Socio-demographic variables of the sample.

Variable	*n*	%
Age group		
Young adult (18–25 years)	134	43.4
Adult (26–44 years)	136	44.0
Middle-aged adult (45–60 years) Gender	39	12.6
Women	148	46.8
Men	165	53.2
Occupation		
Student	125	40.3
Worker	133	42.9
Self-employed	26	8.4
Unemployed	17	5.5
Householder/housewife	2	0.6
Other	7	2.3
Level of education		
Early childhood education	1	0.3
Primary education	4	1.3
Secondary education	101	31.6
Higher education	207	66.8
Marital status		
Singled	145	45.5
Married	162	52.6
Divorced	5	1.6
Other	1	0.3
Children		
Yes	64	20.6
No	249	79.4
Use smartphone in bed		
Yes	247	78.7
No	66	21.3
Time spent using a smartphone in bed		
Less than 15 min	42	16.2
Between 16 and 30 min	90	33.2
Between 31 and 60 min	89	34.0
More than 60 min	41	16.6
Smartphone activity in bed		
Calling	3	1.1
Texting	15	5.7
Internet browsing	59	22.4
Social networks	89	33.8
Multimedia entertainment	82	31.2
Videogames	4	1.5
Other	11	4.2

**Table 2:** Descriptive statistics of the study variables.

Variable	*M*	*SD*
1. Sleep quality (PSQI)	7.5	3.6
2. Problematic smartphone use (SAS-SV)	29.1	10.1
3. Bedtime procrastination (BPS)	29.1	7.3
4. Years with a smartphone	12.0	4.3
5. Daily hours of smartphone use	5.0	2.8
6. Days per week using the smartphone in bed	5.9	1.9
7. Time spent using a smartphone in bed	2.5	1.0

**Table 3:** Pearson’s correlation coefficients of the study variables.

Variable		1	2	3	4	5	6	7
1. Sleep quality (PSQI)	Coef.	—						
	*p*							
2. Problematic smartphone use (SAS-SV)	Coef.	0.160 **	—					
	*p*	0.005						
3. Bedtime procrastination (BPS)	Coef.	0.298 ***	0.363 ***	—				
	*p*	0.000	0.000					
4. Years with a smartphone	Coef.	−0.065	−0.034	−0.140 *	—			
	*p*	0.254	0.547	0.014				
5. Daily hours of smartphone use	Coef.	0.222 **	0.358 ***	0.186 **	−0.040	—		
	*p*	0.000	0.000	0.001	0.487			
6. Days per week of smartphone use in bed	Coef.	−0.006	0.201 **	0.175 *	−0.017	0.183 ***	—	
	*p*	0.922	0.001	0.005	0.784	0.004		
7. Time spent using a smartphone in bed	Coef.	0.173 **	0.286 ***	0.347 ***	−0.172 **	0.275 ***	0.288 ***	—
	*p*	0.005	0.000	0.000	0.006	0.000	0.000	
8. Age	Coef.	−0.063	−0.202 ***	−0.229 ***	0.548 ***	−0.171 **	−0.230 ***	−0.221 ***
	*p*	0.268	0.000	0.000	0.000	0.003	0.000	0.000

Note: Bilateral significance, * *p* < 0.05, ** *p* < 0.01, *** *p* < 0.001.

**Table 4:** Descriptive statistics and gender differences.

Variable	Men		Women		*t*	*gl*	*p*	Cohen’s *d*
	M	SD	M	SD				
1. Sleep quality (PSQI)	7.5	3.6	7.6	3.6	0.210	308	0.834	0.02
2. Problematic smartphone use (SAS-SV)	27.4	9.9	31.0	10.0	3.248	308	0.001	0.36
3. Bedtime procrastination (BPS)	28.7	7.1	29.5	7.5	1.031	308	0.303	0.11
4. Years with a smartphone	12.7	4.3	11.3	3.8	−2.311	306	0.021	−0.26
5. Daily hours of smartphone use	4.7	2.4	5.8	3.2	3.115	306	0.002	0.35
6. Days per week using the smartphone in bed	6.1	1.8	6.0	1.7	0.729	253	0.467	0.09
7. Time spent using a smartphone in bed	2.4	0.9	2.6	0.9	1.740	257	0.083	0.22

Note: Homogeneity of variances tested by Levene’s test (*p* > 0.05).

**Table 5:** One-way ANOVA for the variables under study according to age groups.

Variable	Young Adults (18–25 Years)		Adults (26–44 Years)		Middle-Aged Adults (45–60 Years)		*F*_(2, 306)_	*p*	η2
	*M*	*SD*	*M*	*SD*	*M*	*SD*			
Sleep quality (PSQI)	7.7	3.6	7.5	3.5	6.9	3.9	0.60	0.548	0.00
Problematic smartphone use (SAS-SV)	31.6	9.5	27.6	10.3	25.4	9.3	8.73	<0.001	0.05
Bedtime procrastination (BPS)	30.7	7.6	28.3	7.0	26.2	6.3	7.15	0.001	0.05

Note: Homogeneity of variances tested by Levene’s test (*p* > 0.05).

**Table 6:** Bonferroni post hoc contrasts between age groups for the PSU and bedtime procrastination variables.

Variable	Comparison	Mean Diff.	*SD*	*p*	95% CI	
					Lower Limit	Upper Limit
Problematic smartphone use (SAS-SV)	YA vs. A	4.0	1.2	0.003	1.12	6.90
	YA vs. MAA	6.2	1.8	0.002	1.92	10.55
	A vs. MAA	2.2	1.8	0.651	−2.09	6.52
Bedtime procrastination (BPS)	YA vs. A	2.3	0.9	0.022	0.26	4.47
	YA vs. MAA	4.4	1.3	0.002	1.30	7.60
	A vs. MAA	2.1	1.3	0.333	−1.06	5.23

Note: YA = young adults (18–25 years), A = adults (26–44 years), MAA = middle-aged adults (45–60 years).

**Table 7:** Mediation analysis of the model.

Effect	*B*	*SE (B)*	*t*	*p*	95% CI	
					Lower Limit	Upper Limit
a: PSU → BP	0.26	0.03	6.83	<0.001	1.55	3.20
b: BP → PSQ	0.14	0.03	4.75	<0.001	0.11	0.31
c (total): PSU → PSQ	0.06	0.02	2.84	0.005	−0.02	1.47
c’ (direct): PSU → PSQ	0.02	0.02	1.01	0.312	−0.52	1.00
a*b (indirect): PSU → BP → PSQ	0.04				0.02	0.05

Note: PSU = problematic smartphone use; BP = bedtime procrastination; PSQ = poor sleep quality.

## Data Availability

The data that support the findings of this study are available upon request from the corresponding author.
